# WBRT Plus SIB in Patients with Brain Metastases: Association with Longer Overall Survival and Safety Outcomes in a Single-Center Retrospective Analysis

**DOI:** 10.3390/cancers18203320

**Published:** 2026-10-15

**Authors:** Zibo Tang, Guixiang Liao, Juan Fan

**Affiliations:** Department of Radiation Oncology, Shenzhen People’s Hospital (The First Affiliated Hospital, Southern University of Science and Technology, The Second Clinical Medical College, Jinan University), Shenzhen 518020, China

**Keywords:** brain metastases, whole-brain radiotherapy, simultaneous integrated boost, radiation-induced toxicity

## Abstract

Objective: To compare the efficacy, survival benefits, and safety of whole-brain radiotherapy (WBRT) alone versus WBRT combined with simultaneous integrated boost (WBRT+SIB) for patients with brain metastases. Methods: A total of 100 patients treated from 2017 to 2022 were retrospectively analyzed. Tumor response was assessed per RECIST 1.1. Survival differences were compared using the Kaplan–Meier method and log-rank test. Multivariate Cox regression and subgroup analyses were performed to evaluate the prognostic value and stability of WBRT+SIB. Results: The WBRT+SIB group exhibited numerically higher objective response and disease control rates without statistical significance. With a median follow-up of 31.0 months, WBRT+SIB significantly prolonged median overall survival (11.0 vs. 8.0 months, *p* = 0.011) and improved 1-, 2-, and 3-year survival rates. After adjusting for multiple confounders, WBRT+SIB independently reduced mortality risk (HR = 0.51, 95% CI: 0.27–0.97, *p* = 0.039). Subgroup analyses were exploratory; therefore, consistent survival advantages across strata cannot be confirmed. No significant differences in acute, subacute, or late neurotoxicity, including radiation-induced brain necrosis, were found between the two groups. Conclusions: WBRT+SIB was associated with longer overall survival compared with WBRT alone, without a significant increase in the incidence of radiotherapy-related adverse events. Although ORR and DCR were numerically higher in the WBRT+SIB group, these differences were not statistically significant. Given the limitations of this exploratory study, these findings should be considered hypothesis-generating and require validation in prospective randomized trials.

## Simple Summary

This retrospective study compared whole-brain radiotherapy (WBRT) alone with WBRT plus simultaneous integrated boost (WBRT+SIB) in 100 patients with brain metastases treated between 2017 and 2022. Although objective response and disease control rates were numerically higher with WBRT+SIB, these differences did not reach statistical significance. WBRT+SIB significantly prolonged median overall survival and independently reduced the risk of death, with consistent benefits observed across subgroups. The incidence of acute, subacute, and late neurotoxicity, including radiation-induced brain necrosis, was comparable between the two groups. These findings suggest that WBRT+SIB may confer a survival benefit without significantly increasing radiotherapy-related adverse events.

## 1. Introduction

In the course of cancer progression, 20–40% of patients develop brain metastases (BM) [1,2,3]. Through clinical examinations, imaging diagnoses, or autopsies, 70–80% of these cases are confirmed as multiple brain metastases [4,5]. The median natural survival time of patients with brain metastases does not exceed 3 months [6,7]. Whole brain radiotherapy (WBRT) can extend the median survival time from 1–2 months to 3–6 months [8,9]. However, WBRT is limited by its potential to induce neurological damage, and the total dose generally does not exceed 40 Gy. Borgelt et al. [10] found that there was no significant difference in the median overall survival among intracranial radiotherapy regimens of 20 Gy/5 fractions, 30 Gy/10 fractions, 30 Gy/15 fractions, 40 Gy/15 fractions, and 40 Gy/20 fractions. The National Comprehensive Cancer Network (NCCN) recommends 37.5 Gy in 15 fractions or 30 Gy in 10 fractions as the standard WBRT regimen.

With the widespread implementation of intensity-modulated radiotherapy (IMRT), the application of IMRT for simultaneous intensity-boosted irradiation (SIB) of metastatic lesions during WBRT has improved the local control of metastases and emerged as an alternative treatment modality [11,12]. Nevertheless, high-level clinical evidence for WBRT combined with SIB remains scarce.

Therefore, a retrospective study was carried out to analyze patients with two or more metastatic lesions who underwent either WBRT or WBRT+SIB at our hospital from December 2017 to December 2022. This study aims to investigate the influence of these two radiotherapy approaches on tumor local control and patient prognoses, thereby providing a reference for treatment selection in patients with brain metastases.

## 2. Materials and Methods

### 2.1. Case Selection

This retrospective investigation encompassed patients diagnosed with brain metastases who received treatment in the Radiation Oncology Department of our hospital from December 2017 to December 2022. Patient demographic information, details of clinical management, and radiotherapy plans were retrieved from the hospital’s medical record system. The diagnosis of primary tumors was established based on pathological confirmation of extracranial lesions, whereas intracranial lesions were diagnosed through contrast-enhanced computed tomography (CT) or magnetic resonance imaging (MRI) of the brain.

The inclusion criteria were as follows: (1) A Karnofsky Performance Status (KPS) score of 60 or higher; (2) A life expectancy of at least 1 month; (3) An age of 18 years or older; (4) The presence of at least one measurable lesion prior to radiotherapy; (5) Adequate hematologic function, specifically a neutrophil count of 1.5 × 10^9^/L or higher, a hemoglobin level of 90 g/L or higher, and a platelet count of 100 × 10^9^/L or higher.

The exclusion criteria were: (1) Total loss of sensory function; (2) Severe impairment of consciousness (stupor or coma); (3) Psychologically diagnosed disorders or other severe chronic conditions, such as uncontrolled diabetes, severe heart disease, and hypertension; (4) Pregnant or lactating female patients; (5) Patients with surgically resected solitary intracranial metastatic lesions; (6) Patients with leptomeningeal metastasis or cerebrospinal fluid dissemination.

This study protocol was approved by the Ethics Committee of our institution.

### 2.2. Case Grouping and Radiotherapy Regimens

Patients were divided into two groups based on irradiation modality: WBRT and WBRT+SIB. For patients with 1–3 brain metastases, WBRT+SIB was offered instead of SRS when lesions were unsuitable for SRS (size > 3 cm, eloquent locations, significant edema, prior SRS, or logistical/economic barriers). Patients with 1–3 metastases who met SRS criteria received SRS and were excluded.

Because this was a retrospective study, treatment allocation was not randomized. The choice of WBRT alone versus WBRT+SIB was determined by the treating radiation oncologist and multidisciplinary team based on clinical judgment. Factors considered included the number, size/volume, and location of brain metastases; presence and extent of peritumoral edema; KPS/performance status; extracranial disease burden and control; expected survival; prior surgery, radiotherapy, or systemic therapy; availability and type of systemic therapy; and practical or patient-related considerations such as treatment duration and ability to tolerate immobilization. In general, WBRT alone tended to be selected for patients with multiple, relatively small brain metastases; lesions located adjacent to critical structures such as the brainstem or optic chiasm; poorer performance status; higher extracranial tumor burden; and shorter expected survival. By contrast, WBRT+SIB tended to be selected for patients with fewer but larger metastases; adequate performance status to tolerate treatment intensification; and more favorable expected survival. These were general clinical tendencies rather than strict selection criteria.

Patients were divided into WBRT alone (*n* = 60) and WBRT+SIB (*n* = 40). Prior to radiotherapy planning, all patients underwent 1.5T MRI with 3 mm slice thickness for CT-MRI fusion. Simulation CT scans were acquired with 3 mm slice thickness, and plans were designed using MONACO.

Target volume definitions and dose distribution for both groups:

WBRT group: The clinical target volume (CTV) encompassed all brain tissue (from the skull base to the C2 level). The planned target volume (PTV) extended 3 mm beyond the CTV. The prescribed dose was 30–40 Gy in 10–20 fractions over 2–4 weeks, with 95% of the PTV receiving the prescribed dose. The BED and EQD2 values for the different radiotherapy regimens are presented in the table below, calculated with an α/β ratio of 10 Gy.

WBRT-SIB group: On CT-MRI fusion images, the gross tumor volume (GTVn) of intracranial metastases was delineated, with a 3 mm extension forming the planned target volume (PTVtn). Multiple metastases were labeled as PTVt1, PTVt2, …, PTVn. The clinical target volume (CTV) and planned target volume (PTV) were identical to the WBRT group. Critical structures included the brainstem, optic nerves, optic chiasm, lens, and eyeballs. Prescribed doses were 95% PTVtn at 36–60 Gy in 10–20 fractions over 2–4 weeks, and 95% PTV at 30–40 Gy in 10–20 fractions over 2–4 weeks (Supplementary Materials Table S1).

### 2.3. Evaluation Criteria

Immediate efficacy was assessed at the first follow-up MRI (4–6 weeks post-radiotherapy) using RECIST 1.1 criteria, with responses categorized as complete response (CR), partial response (PR), stable disease (SD), or progressive disease (PD). Objective response rate (ORR) was defined as (CR + PR)/total cases, and disease control rate (DCR) as (CR + PR + SD)/total cases. Overall survival (OS) was measured from treatment initiation to death or last follow-up.

### 2.4. Safety Analysis

Adverse events were retrospectively identified from electronic medical records, radiotherapy records, inpatient and outpatient notes, imaging reports, laboratory results, and medication records. Only events documented during follow-up and considered possibly related to radiotherapy were included. Toxicities were graded using the RTOG/EORTC criteria. Acute toxicity was defined as events occurring during radiotherapy through 90 days after completion of radiotherapy; subacute toxicity as events occurring 3–6 months after radiotherapy; and late toxicity as events occurring >6 months after radiotherapy. Patients who deteriorated before imaging were considered non-evaluable and were excluded from the primary analysis; in a sensitivity analysis, they were counted as having progressive disease (PD). Patients lost to follow-up were excluded.

Radiation-induced brain necrosis was diagnosed when MRI demonstrated a new or enlarging contrast-enhancing lesion within the irradiated volume, accompanied by surrounding vasogenic edema, supported by advanced imaging findings (decreased perfusion, elevated choline/creatine ratio on MRS, or increased amino acid PET uptake), and after exclusion of tumor progression. Histopathological confirmation was used when available.

### 2.5. Statistical Methods

Data analysis was performed using SPSS 22.0. Comparisons between the WBRT and WBRT+SIB groups were conducted using the chi-square test. Survival analysis was performed using the Kaplan–Meier method, and survival curves were compared using the log-rank test. Cox proportional hazards models were used to analyze the association between treatment modalities and mortality risk. Model 1 adjusted for age and sex, while Model 2 further adjusted for age, sex, temozolomide, KPS score, time from diagnosis to treatment (the date of histopathological diagnosis of the primary tumor to the date of initiation of radiotherapy for brain metastases), stability of primary lesion, number of intracranial metastases, and primary lesion status. Hazard ratios (HR) and their 95% confidence intervals (CI) were calculated. Subgroup analyses were conducted based on age, sex, temozolomide, KPS score, time from diagnosis to treatment, stability of primary lesion, number of intracranial metastatic lesions, and primary lesion status. Adjusted factors were consistent with Model 2 but excluded stratification factors. Subgroup analyses were exploratory and not used for statistical inference regarding treatment efficacy. Statistical significance was defined as two-sided *p* < 0.05.

## 3. Results

### 3.1. General Data

From December 2017 to December 2022, 152 patients with brain metastases were identified in our hospital’s case system. After excluding 52 cases based on inclusion/exclusion criteria, 100 patients were enrolled for analysis. The WBRT group comprised 60 patients, while the WBRT+SIB group included 40 patients (Table 1). The baseline characteristics of the two groups are summarized in Table 1. There were no significant intergroup differences in gender, age, temozolomide application, KPS score, primary disease stability, number of brain metastases, and primary tumor type. The time interval from diagnosis to treatment differed significantly between groups (*p* = 0.041).

### 3.2. Short-Term Efficacy

The WBRT group achieved 1 complete response (CR), 27 partial responses (PR), 16 stable disease (SD) cases, and 13 progressive disease (PD) cases; the WBRT+SIB group achieved 3 CR, 18 PR, 9 SD, and 6 PD cases (Table 2). The ORR was 48.3% in the WBRT group and 57.5% in the WBRT+SIB group. The DCR was 76.7% and 82.5% for the WBRT group and WBRT+SIB group, respectively. Although the WBRT+SIB group exhibited numerically superior ORR and DCR, there was no significant difference in ORR or DCR between the two groups.

### 3.3. Survival Outcomes

**Figure 1:**
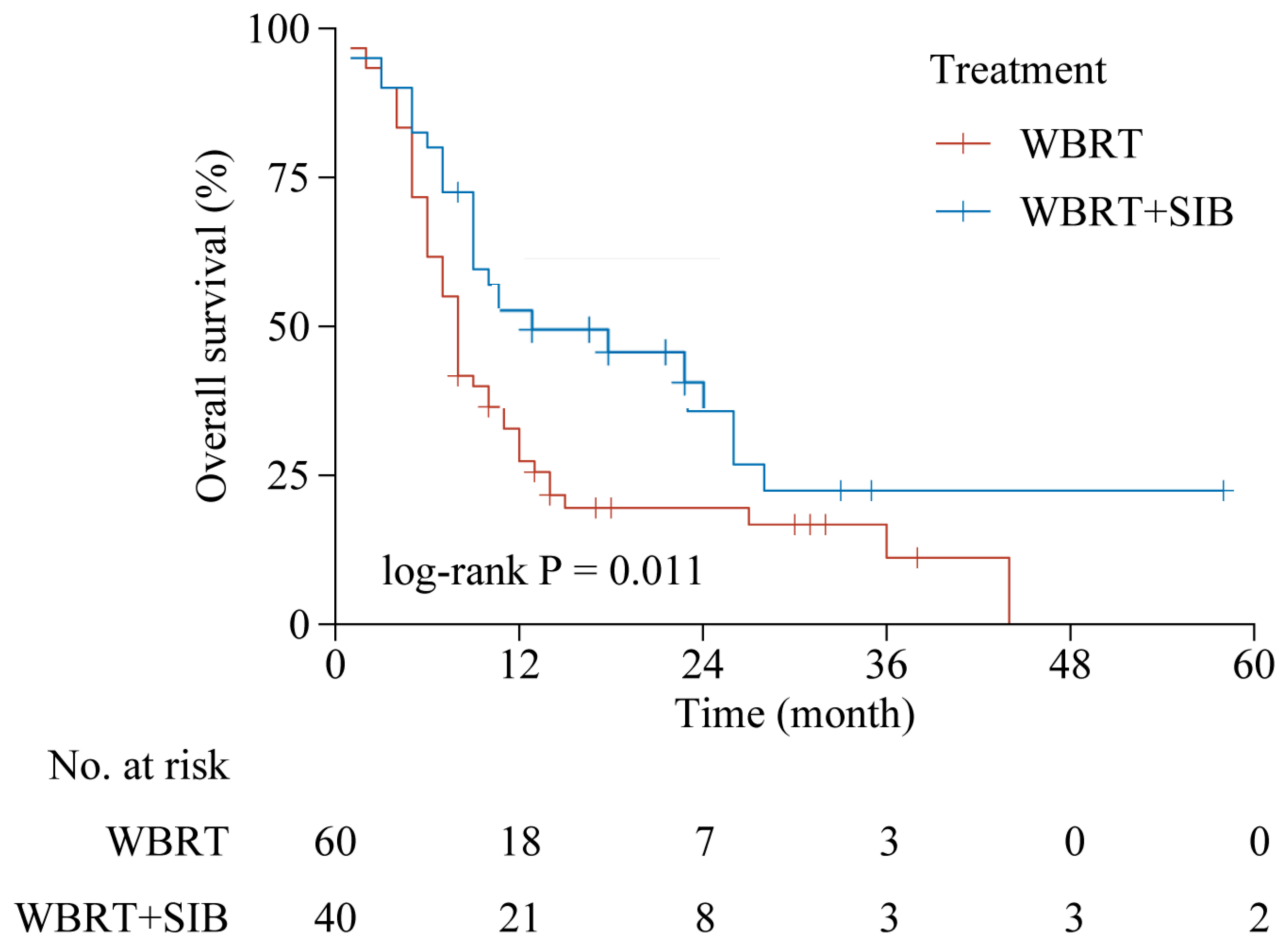
Kaplan–Meier curves of overall survival according to treatment. Abbreviations: WBRT, Whole-brain radiotherapy; SIB, Simultaneous integrated boost. The number of patients in each group at every 12-month interval is indicated. *p* values were calculated by a log-rank test.

The median follow-up duration for all patients was 31.0 months (range: 1–82 months). At the time of last follow-up, 24 patients were alive and 76 had died. The median overall survival (OS) was 8.0 months (95% CI: 7.0–11.0 months) in the WBRT group and 11.0 months (95% CI: 8.0–13.0 months) in the WBRT+SIB group. The 1-, 2-, and 3-year overall survival rates were 27.3%, 19.4%, and 11.1% in the WBRT group, and 51.4%, 35.7%, and 22.3% in the WBRT+SIB group, respectively. Survival curves between the two groups showed statistically significant differences (*p* = 0.011), as shown in Figure 1.

Cox proportional hazards models analyzed the association between treatment modality and overall survival. Model 1, adjusted for age and sex, showed a lower risk of death in the WBRT+SIB group compared to the WBRT group (HR = 0.48, 95% CI: 0.29–0.78, *p* = 0.003). After adjusting for age, sex, temozolomide, KPS score, time from diagnosis to treatment, primary lesion stability, number of intracranial metastases, and primary lesion status in Model 2, the WBRT+SIB group exhibited a lower risk of death compared to the WBRT group (HR = 0.51, 95% CI: 0.27–0.97, *p* = 0.039). See Table 3.

### 3.4. Comparison of Treatment-Related Toxicities Between WBRT and WBRT+SIB

The incidence of treatment-related toxicities is summarized in Table 4. No statistically significant differences were observed between the two groups for any of the reported symptoms, across all grade categories (all *p* values > 0.05, Fisher’s exact test). Specifically, for Grade 1-2 adverse events, the rates were comparable between the WBRT and WBRT+SIB groups for headache (45.0% vs. 55.0%, *p* = 0.322), nausea/vomiting (23.3% vs. 27.5%, *p* = 0.639), hearing loss (3.3% vs. 5.0%, *p* = 0.646), fatigue (55.0% vs. 50.0%, *p* = 0.685), memory loss (30.0% vs. 35.0%, *p* = 0.595), and speech disorder (5.0% vs. 10.0%, *p* = 0.432). For severe adverse events (SAE, Grade 3-5), the incidence remained low in both groups, with no significant differences detected for headache, nausea/vomiting, fatigue, or memory loss. No Grade 3-5 events were reported for hearing loss or speech disorder in either group. The incidence of grade 1–2 radiation-induced brain necrosis was 3.3% in the WBRT group and 5.0% in the WBRT+SIB group (*p* = 0.640). Grade 3–5 radiation-induced brain necrosis occurred in 1.7% of WBRT patients and 5.0% of WBRT+SIB patients (*p* = 0.558). The overall incidence of any-grade radiation-induced brain necrosis was 5.0% versus 10.0%, respectively (*p* = 0.439). No statistically significant difference in radiation-induced brain necrosis was observed between the two groups.

These findings indicate that the addition of a simultaneous integrated boost to whole-brain radiotherapy was not associated with a statistically significant increase in acute or subacute toxicities. Although the incidence of radiation-induced brain necrosis was numerically higher in the WBRT+SIB group, no significant difference in late neurotoxicity was observed between the two groups. However, given the relatively short survival of many patients, late toxicity could not be fully assessed; therefore, these results should be interpreted with caution.

### 3.5. Subgroup Analysis

Stratified analysis was performed based on age, gender, concomitant temozolomide use, KPS score, time from diagnosis to treatment, stability of primary lesion, number of intracranial metastases, and primary lesion. After adjusting for potential confounders, the HR direction remained consistent across all strata, with no statistically significant differences between the WBRT+SIB and WBRT groups (all *p* > 0.05; Table 5). These subgroup analyses should be considered exploratory. Although the direction of the hazard ratios generally favored WBRT+SIB, most subgroup estimates had wide confidence intervals that crossed 1.0, and interaction *p* values were not calculated. Therefore, no definitive conclusions regarding treatment-effect heterogeneity across strata can be drawn, and these findings should be interpreted with caution.

## 4. Discussion

Brain metastases are common in the natural course of solid tumors, and despite aggressive treatment, their prognosis remains poor [2,3]. Due to the blood–brain barrier, most chemotherapy regimens are ineffective. WBRT serves as an important palliative treatment for patients with brain metastases, offering symptomatic relief, improved quality of life, and prolonged survival while reducing recurrence risk [13]. WBRT can extend overall survival by 4 to 6 months in patients with brain metastases [12,14]. Studies indicate that local control rates in patients with brain metastases correlate positively with the total radiation dose. It is generally recommended that the maximum WBRT dose be limited to below 40 Gy, as higher doses may increase biological toxicity to brain parenchyma and even cause irreversible brain tissue damage [15,16]. The currently recommended WBRT fractionation schemes are 30 Gy in 10 fractions or 37.5 Gy in 15 fractions. Previous studies have demonstrated a high intracranial recurrence rate in brain metastasis patients following WBRT alone. For patients with solitary or <3 metastatic lesions, combining WBRT with surgical resection of the metastatic focus, stereotactic radiosurgery (SRS), or three-dimensional conformal radiotherapy (3DCRT) yields a median overall survival (OS) superior to WBRT alone [17,18,19]. However, administering local boost irradiation after WBRT increases radiation doses to surrounding normal brain tissue and prolongs treatment duration. Consequently, researchers have extensively explored treatment modalities that neither significantly elevate brain tissue radiation exposure nor substantially extend treatment time.

Synchronous Intensity-Modulated Radiation Therapy (SIB-IMRT) builds upon IMRT technology by simultaneously delivering locally increased doses to the tumor target volume. This approach enhances both the single-fraction dose and total dose to the target, thereby increasing the biological effect of radiotherapy and significantly boosting tumor cell killing [12,20]. SIB-IMRT can simultaneously achieve treatment objectives for two or more target volumes, offering advantages such as high target dose, uniform dose distribution, enhanced biological effect, and reduced radiation exposure to normal tissues [21]. Dong et al. [20] reported that patients with 1–7 brain metastases from non-small cell lung cancer treated with WBRT+SIB (52.5 Gy/15 fractions) achieved a median overall survival (OS) of 20.0 months with tolerable toxicity. The median OS in the WBRT+SIB group of this study was 11.0 months, slightly lower than that reported by DONG et al. [20], possibly due to the inclusion of multiple primary tumor types in this study. Additionally, KIM et al. [22] reported a 1-year intracranial lesion control rate of 67% in NSCLC patients with brain metastases treated with WBRT+SIB. In our study, the DCR in the WBRT+SIB group was 82.5%, higher than those reported by Kim et al. [22]. However, direct comparison between these values should be made with caution.

SIB-IMRT is used to treat brain metastases, with treatment objectives extending beyond controlling metastatic lesions to also managing disseminated brain metastases, including both macroscopic and microscopic lesions. For instance, when WBRT is administered at 37.5 Gy in 15 fractions, the single-fraction dose to brain metastases reaches 3.6–4 Gy, while doses to critical structures such as the brainstem and optic nerves remain strictly confined within dose limits. The present study showed numerically higher ORR and DCR in the WBRT+SIB group relative to the WBRT group, although these differences did not reach statistical significance. No patients interrupted or discontinued radiotherapy because of SIB-related adverse events. Survival analysis demonstrated favorable overall survival for patients receiving WBRT+SIB. After adjustment for multiple clinical confounders, WBRT+SIB remained independently associated with reduced mortality risk.

Several limitations of this retrospective single-center study should be acknowledged. First, baseline imbalance existed between groups, particularly for the time interval from diagnosis to treatment. Despite multivariate Cox regression adjustment, residual confounding and selection bias cannot be completely eliminated. Accordingly, our results are hypothesis-generating and cannot establish definitive causal conclusions. Second, although radiation-induced brain necrosis was assessed in our cohort, formal systematic evaluation of long-term neurocognitive decline was lacking. Given the limited sample size, stratified analyses by individual primary tumor subtypes were not feasible. Consistent with published reports [23,24,25], we observed no statistically significant excess of radiotherapy-related toxicities in the WBRT+SIB arm. Still, prospective data are required to validate the safety profile of WBRT+SIB. Third, the external validity of our findings is restricted to patients who are eligible for stereotactic radiosurgery (SRS). In patients with oligometastases (1-3 brain metastases), WBRT+SIB was adopted when SRS was inappropriate owing to lesion size, eloquent location, perilesional edema or practical constraints. Therefore, our data do not support the conclusion that WBRT+SIB outperforms SRS for SRS-eligible oligometastatic brain metastases. Fourth, several important prognostic factors could not be comprehensively assessed because of the retrospective design. Data on extracranial disease burden and detailed systemic therapy were incomplete. Specifically, molecular testing and systemic treatment data were largely unavailable. Temozolomide was the only consistently recorded systemic agent and was used concurrently with radiotherapy only in patients with non-small cell lung cancer and triple-negative breast cancer, whereas immunotherapy and targeted therapy were not administered concurrently with radiotherapy. These missing data may introduce confounding and limit the generalizability of our findings. Therefore, our results should be interpreted with caution and warrant prospective validation with complete molecular and systemic treatment information.

In conclusion, WBRT+SIB yields promising survival benefits without significantly increasing radiotherapy-associated adverse events in selected patients with brain metastases. Nevertheless, owing to the retrospective nature and inherent selection bias of this study, these observations should be interpreted cautiously. Prospective randomized controlled trials are warranted to reliably compare efficacy, long- term neurocognitive function and quality of life between WBRT+SIB and WBRT alone. Further investigations are also needed to clarify the optimal integration of radiotherapy with chemotherapy, targeted agents or immunotherapy for brain metastases.

## 5. Conclusions

In this retrospective single-center study, WBRT+SIB appeared to be associated with improved overall survival and lower mortality risk compared with WBRT alone in selected patients with brain metastases. However, given the retrospective design and potential selection bias, these findings should be considered hypothesis-generating and may have limited generalizability. Prospective randomized trials are needed to clarify efficacy, safety, long-term neurocognitive outcomes, and the optimal combination with systemic therapy.

## Figures and Tables

**Table 1:** Baseline characteristics according to treatment.

Characteristic	WBRT (*n* = 60)	WBRT+SIB (*n* = 40)	χ^2^	*p* Value
Gender			0.240	0.624
Female	30 (50.0%)	18 (45.0%)		
Male	30 (50.0%)	22 (55.0%)		
Age, year			2.554	0.110
<60	41 (68.3%)	21 (52.5%)	45.89 ± 7.32	
≥60	19 (31.7%)	19 (47.5%)	65.82 ± 5.34	
Temozolomide			2.554	0.110
Yes	19 (31.7%)	19 (47.5%)		
No	41 (68.3%)	21 (52.5%)		
KPS score			0.007	0.933
≤70	37 (61.7%)	25 (62.5%)		
>70	23 (38.3%)	15 (37.5%)		
Time from diagnosis to treatment, months			4.167	0.041
≤12	35 (58.3%)	15 (37.5%)	6.00 ± 3.60	
>12	25 (41.7%)	25 (62.5%)	40.57 ± 29.51	
Whether the primary disease is stable			0.063	0.802
Yes	24 (40.0%)	15 (37.5%)		
No	36 (60.0%)	25 (62.5%)		
Number of brain metastases			0.073	0.787
1~2	18 (30.0%)	11 (27.5%)		
≥3	42 (70.0%)	29 (72.5%)		
Primary			0.246	0.884
Lung	30 (50.0%)	22 (55.0%)		
Breast	18 (30.0%)	11 (27.5%)		
Others	12 (20.0%)	7 (17.5%)		

Abbreviations: KPS, Kanofsky performance score; WBRT, Whole-brain radiotherapy; SIB, Simultaneous integrated boost. Data are *n* (%), unless otherwise specified.

**Table 2:** Comparison of the secondary evaluation indexes of the two groups of patients.

Response	WBRT (*n* = 57)	WBRT+SIB (*n* = 36)	χ^2^	*p* Value
CR	1	3	—	—
PR	27	18	—	—
SD	16	9	—	—
PD	13	6	—	—
ORR	48.3%	57.5%	0.808	0.369
DCR	76.7%	82.5%	0.588	0.443

Abbreviations: CR, Complete response; PR, Partial response; SD, Stable disease; PD, Progressive disease; ORR, Overall response rate; DCR, Disease control rate; WBRT, Whole brain radiotherapy; SIB, Simultaneous integrated boost.

**Table 3:** Association of treatment with overall survival in patients by Cox proportional hazards regression.

Treatment	No. of Death/Total	Model 1 ^a^		Model 2 ^b^	
		HR (95% CI)	*p* Value	HR (95% CI)	*p* Value
WBRT	50/60	1.00		1.00	
WBRT+SIB	26/40	0.48 (0.29–0.78)	0.003	0.51 (0.27–0.97)	0.039

Abbreviations: HR, hazard ratio; CI, confidence interval; WBRT, Whole-brain radiotherapy; SIB, Simultaneous integrated boost. ^a^ Model 1 adjusted for gender and age. ^b^ Model 2 adjusted for gender, age, temozolomide, KPS score, time from diagnosis to treatment, whether the primary disease is stable, number of brain metastases, and primary.

**Table 4:** Treatment-Related Adverse Events.

Symptom	WBRT (*n* = 60)	WBRT+SIB (*n* = 40)	*p* Value
Headache			
Grade 1–2	27	22	0.322
Grade 3–5	4	2	1
Nausea/Vomiting			
Grade 1–2	14	11	0.639
Grade 3–5	2	3	0.380
Hearing loss			
Grade 1–2	2	2	0.646
Grade 3–5	0	0	-
Fatigue			
Grade 1–2	33	20	0.685
Grade 3–5	2	2	0.646
Memory loss			
Grade 1–2	18	14	0.595
Grade 3–5	6	4	1
Speech Disorder			
Grade 1–2	3	4	0.432
Grade 3–5	0	0	-
Radiation-induced necrosis			
Grade 1–2	2	2	0.439
Grade 3–5	1	2	-

Radiation toxicity (RTOG/EORTC). RTOG, Radiation Therapy Oncology Group; EORTC, European Organization for Research and Treatment of Cancer.

**Table 5:** Association between treatment and overall survival stratified by different factors *.

Subgroup *	No. of Death/Total	HR (95% CI)
Gender		
Female	34/48	0.39 (0.14–1.09)
Male	42/52	0.81 (0.32–2.06)
Age, year		
<60	48/62	0.46 (0.19–1.09)
≥60	28/38	0.95 (0.34–3.25)
Temozolomide		
Yes	27/38	0.65 (0.26–1.63)
No	49/62	0.40 (0.17–1.96)
KPS score		
≤70	53/62	0.53 (0.20–1.40)
>70	23/38	0.61 (0.26–1.46)
Time from diagnosis to treatment, months		
≤12	39/50	0.91 (0.36–2.28)
>12	37/50	0.35 (0.10–1.66)
Whether the primary disease is stable		
Yes	27/39	0.87 (0.63–1.46)
No	49/61	0.56 (0.20–1.44)
Number of brain metastases		
1~2	21/29	0.29 (0.09–1.92)
≥3	55/71	0.73 (0.32–1.66)
Primary		
Lung	41/52	0.48 (0.19–1.83)
Breast	21/29	0.53 (0.22–1.57)
Others	14/19	0.64 (0.24–1.71)

Abbreviation: HR, hazard ratio; CI, confidence interval; KPS, Kanofsky performance score. * Data were fitted by Cox proportional hazards regression models, adjusted for gender, age, temozolomide, KPS score, time from diagnosis to treatment, whether the primary disease is stable, number of brain metastases, and primary disease.

## Data Availability

The datasets that were analyzed and/or generated during this study are available from the corresponding author upon reasonable request.

## Supplementary Materials

The following supporting information can be downloaded at: https://www.mdpi.com/article/10.3390/cancers18203320/s1, Table S1: BED and EQD2 of WBRT/SIB regimens.
