# Supplementary material for: WBRT Plus SIB in Patients with Brain Metastases: Association with Longer Overall Survival and Safety Outcomes in a Single-Center Retrospective Analysis
Source: Cancers (Basel). 2026 Oct 15;18(20):3320. doi: 10.3390/cancers18203320 (PMC13702360; doi:10.3390/cancers18203320)
Supplement: Supplementary file 1 [file cancers-18-03320-s001.zip › cancers-4543185-supplementary.pdf]

Supplementary Materials

WBRT Plus SIB in Patients with Brain Metastases: Association with Longer Overall Survival and Safety Outcomes in a Single-Center Retrospective Analysis

Supplementary Table S1. BED and EQD2 of WBRT/SIB regimens.

Regimen	BED (Gy)	EQD2 (Gy)
WBRT 30 Gy/10 fx	39.00	32.50
WBRT 37.5 Gy/15 fx	46.88	39.06
WBRT 40 Gy/20 fx	48.00	40.00
SIB 36 Gy/10 fx	48.96	40.80
SIB 52.5 Gy/15 fx	70.88	59.06
SIB 60 Gy/20 fx	78.00	65.00
